# Chromosome Doubling-Enhanced Biomass and Dihydrotanshinone I Production in *Salvia miltiorrhiza*, A Traditional Chinese Medicinal Plant

**DOI:** 10.3390/molecules23123106

**Published:** 2018-11-27

**Authors:** Elena Gamboa Chen, Kang-Lun Tsai, Hsiao-Hang Chung, Jen-Tsung Chen

**Affiliations:** 1 Department of Life Sciences, National University of Kaohsiung, Kaohsiung 811, Taiwan; cat790621@yahoo.com.tw (E.G.C.); ktsai7484@gmail.com (K.-L.T.); 2 Department of Horticulture, National Ilan University, Yilan 260, Taiwan; hhchung@niu.edu.tw

**Keywords:** antimitotic agent, direct shoot formation, flow cytometry, polyploidy, salvianolic acid B, tanshinone

## Abstract

The root of Chinese sage (*Salvia miltiorrhiza* Bunge) was regarded as top-grade Chinese medicine two thousand years ago, according to *Shen Nong Materia Medica*. The aim of this study is to develop an easy and reliable means for obtaining tetraploids (4*x* plants) via thidiazuron-induced direct organogenesis in the presence of colchicine. The resulting 4*x* plants showed significantly enhanced agronomic traits, including the size of stomata, leaflet, pollen, and seed as well as shoot length, root diameter, number of leaves, and fresh weight of plant. In addition, an obvious reduction of length to width ratio was found in the 4*x* plants, including stomata, leaflets, pollens, seeds, and roots. The 4*x* ploidy state of the plants was stable as was proved by evaluation of selection indicators as well as consistent ploidy level at 10th generation plantlets and also on 4*x* seedlings obtained via self-pollination. The major bioactive compounds, salvianolic acid B, tanshinone I, tanshinone IIA, dihydrotanshinone I and cryptotanshinone, as well as total tanshinones were determined by high performance liquid chromatography (HPLC). The concentrations of dihydrotanshinone I and total tanshinones in the root extract of the 4*x* plants were significantly higher when compared with the 2*x* plants. This present study developed a simple and efficient system for inducing and subculture of tetrapolids which have stable ploidy level, enhanced growth characteristics as well as the content of dihydrotanshinone I in the root of *S. miltiorrhiza*.

## 1. Introduction

The root of Chinese sage (*Salvia miltiorrhiza* Bunge), also known as Danshen or Tanshen, has been used for thousands of years as a top-grade traditional Chinese medicine since it had been documented in *Shen Nong Materia Medica* [1,2]. The main bioactive compounds in the dried root of Chinese sage are tanshinones and related quinones, which are chiefly used for treating cardiovascular diseases and also heart pain [2,3,4]. More than 30 tanshinones have been isolated from the root of Chinese sage, with the four major tanshinones being, cryptotanshinone (CT), tanshinone I (T-I), tanshinone IIA (T-IIA) and dihydrotanshinone I (DT-I) [2,3]. In addition the phenolic compound, salvianolic acid B (SA-B), accumulates in the root and it has potent therapeutic effects on cardiovascular diseases [2,4,5].

The scientific research on Chinese sage have mainly focusing on in vitro culture techniques, hairy root induction, polyploid breeding, and also biosynthesis of medicinal constituents [1,2,6]. In vitro culture protocols have been established for nutrient feeding, elicitor stimulation and medium optimization, for enhancing tanshinone production [2]. However, only a few protocols were proposed for micropropagation, and the regeneration pathway via adventitious shoot induction [6]. However, these protocols have not been used for successful polyploidy induction in Chinese sage.

The induction of polyploidy is an effective technique for producing novel genotypes and also for enhancing agronomic characteristics, as well as studying plant breeding and genetic mechanisms [7,8,9,10,11]. Explant type and regeneration pathways are critical in polyploidy induction because these factors affect the ratio of pure polyploids to chimeras [7,9,10]. Autotetraploid plants can be induced using colchicine treatment on Chinese sage, and they used seed-derived bud clumps as the explants to induce tetraploids [1]. However, the progeny obtained from sexual propagation are not uniform, limiting the practical application. In Gao et al. the rate of polyploidization was evaluated using time-consuming methods and the accumulation of the two major bioactive compounds, DT-I and SA-B, was not determined [1].

This study establishes a more efficient system for inducing the polyploidy of Chinese sage. First, we used vegetative tissues from clonal plantlets, rather than seed-derived bud clumps, as the explant sources. Second, the polyploids were induced via thidiazuron (TDZ)-enhanced direct organogenesis [12], and in the presence of extremely low dosage of antimitotic agents to avoid abnormality and excess ploidy. Third, the rate of polyploidy was evaluated using flow cytometry instead of chromosome counting and morphological observation to increase efficiency. Fourth, the major bioactive compounds, including SA-B, CT, DT-I, T-I and T-IIA, were all quantified by high performance liquid chromatography (HPLC) in both of the shoot and the root of tetraploids.

## 2. Results

### 2.1. Induction and Identification of Polyploidy

**Figure 1:**
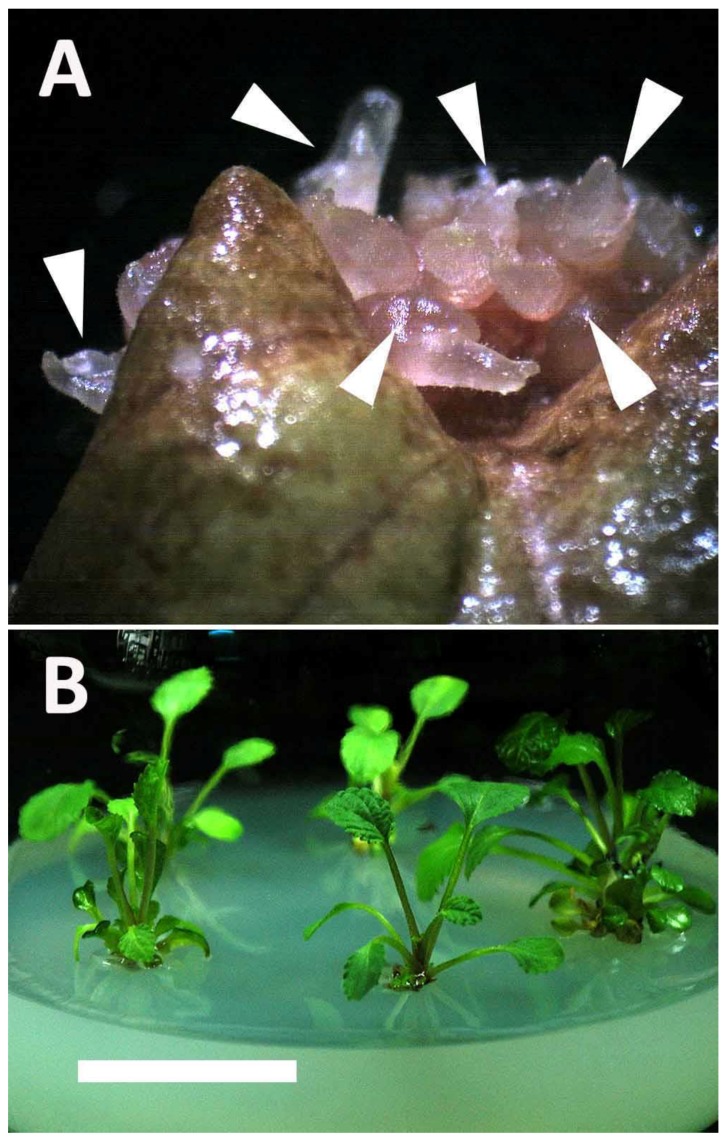
Induction of polyploids via TDZ-induced direct organogenesis from leaf explants of *Salvia miltiorrhiza* on colchicine-containing medium (bar = 5 and 14 mm for Figure 1A,B, respectively). (**A**) Shoot buds (arrowhead) formed directly from the leaf explants after eight weeks of culture. (**B**) Shoot-derived plantlets after 17 weeks of culture.

**Figure 2:**
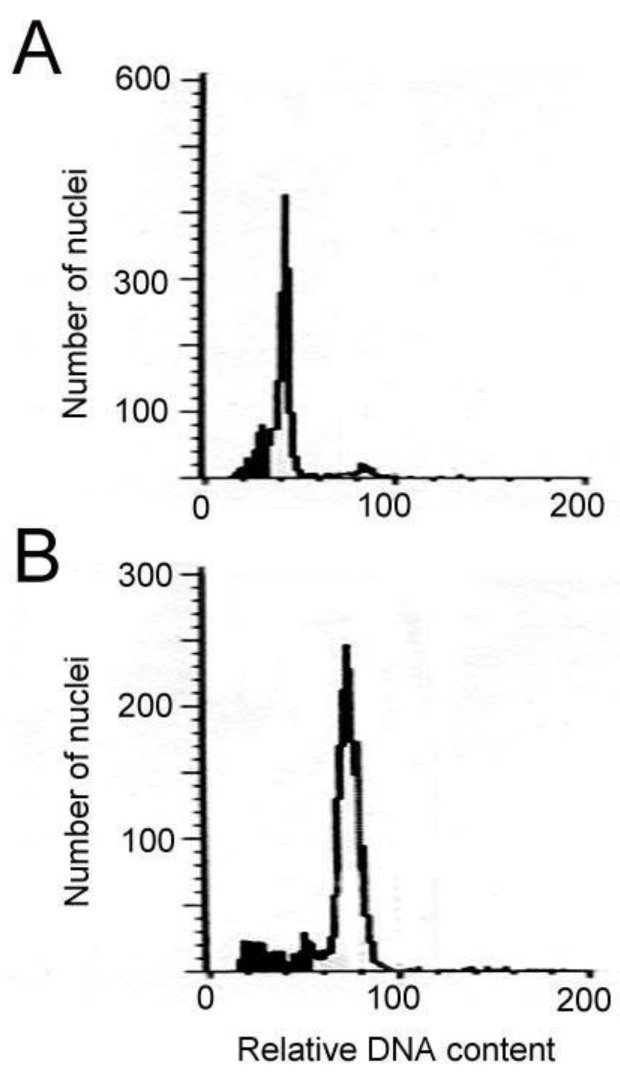
Flow cytometric analysis of the plantlets of *Salvia miltiorrhiza*. (**A**) Diploid. (**B**) Tetraploid.

The formation of shoots from leaf explants was significantly retarded by colchicine (Table 1). After four weeks of culture, small swelling masses began to form, and the parent leaf explants became brown. After an additional four weeks, young shoots developed and the parent leaf explants became necrotic (Figure 1A). These shoots were transferred to a rooting medium supplemented with 0.5 mg/L Indole-3-butyric acid (IBA) for plantlet recovery (Figure 1B). Colchicine at 5, 10, 50, and 100 mg/L totally inhibited shoot formation from the leaf explants regardless of the presence of *N^6^*-benzyladenine (BA) and TDZ. However, adventitious shoots could be obtained from the leaf explants in several treatments, including 0.5 mg/L colchicine plus 0.5 mg/L BA and 0.5, 1, 2, 3 or 4 mg/L colchicine plus 0.5 mg/L TDZ (Table 1). The flow cytometric analysis proved that the tetraploids could be obtained in 4 treatments, and the highest percentages of tetraploids were 39.0% at 0.5 mg/L colchicine plus 0.5 mg/L TDZ (Table 1). There were two types of ploidy that were found in the regenerated plants, the 2*x* and 4*x* (Figure 2). In the presence of 0.5 mg/L TDZ, the 4*x* plants which obtained at 2 mg/L colchicine were selected for all the further tests (i.e., agronomic traits and chemical constituents).

### 2.2. In Vitro Growth of Tetraploids

**Figure 3:**
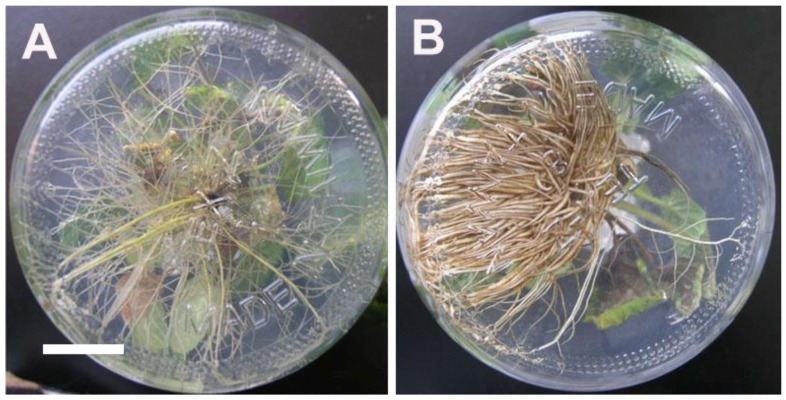
Growth and morphological responses of 2*x* and 4*x* plants of *Salvia miltiorrhiza* in vitro (bar = 2 cm for all figures). (**A**) 2*x* plant. (**B**) 4*x* plant.

**Figure 4:**
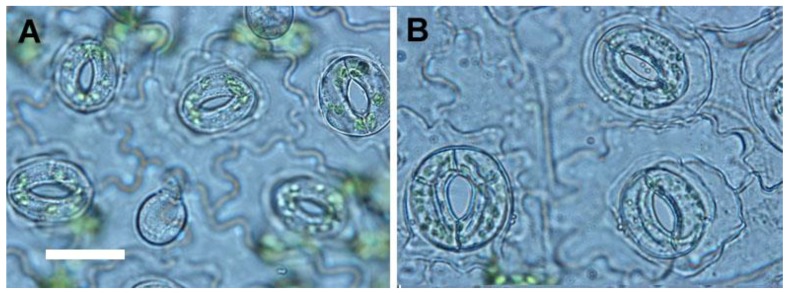
Stomata of 2*x* and 4*x* plants in *Salvia miltiorrhiza* (bar = 15 μm for all figures). (**A**) 2*x* plant. (**B**) 4*x* plant.

In 2.5-month-old in-vitro-grown plantlets, the growth behaviors, including fresh weight, shoot length, number of leaves and root diameter of 4*x* plants were all significantly higher than 2*x* plants (Table 2). By contrast, the root length of 4*x* plants was significantly lower than 2*x* plants (Table 2). The roots of 4*x* plants was darker, shorter and thicker than 2*x* plants (Figure 3). The stomata frequency of 4*x* plants was significantly lower than 2*x* plants, but the stomata length and width of 4*x* plants were significantly larger than 2*x* plants (Table 3, Figure 4). In addition, the size of epidermal cells in 4*x* plants was larger than 2*x* plants (Figure 4).

### 2.3. Growth Characteristics of Ex Vitro Plants

**Figure 5:**
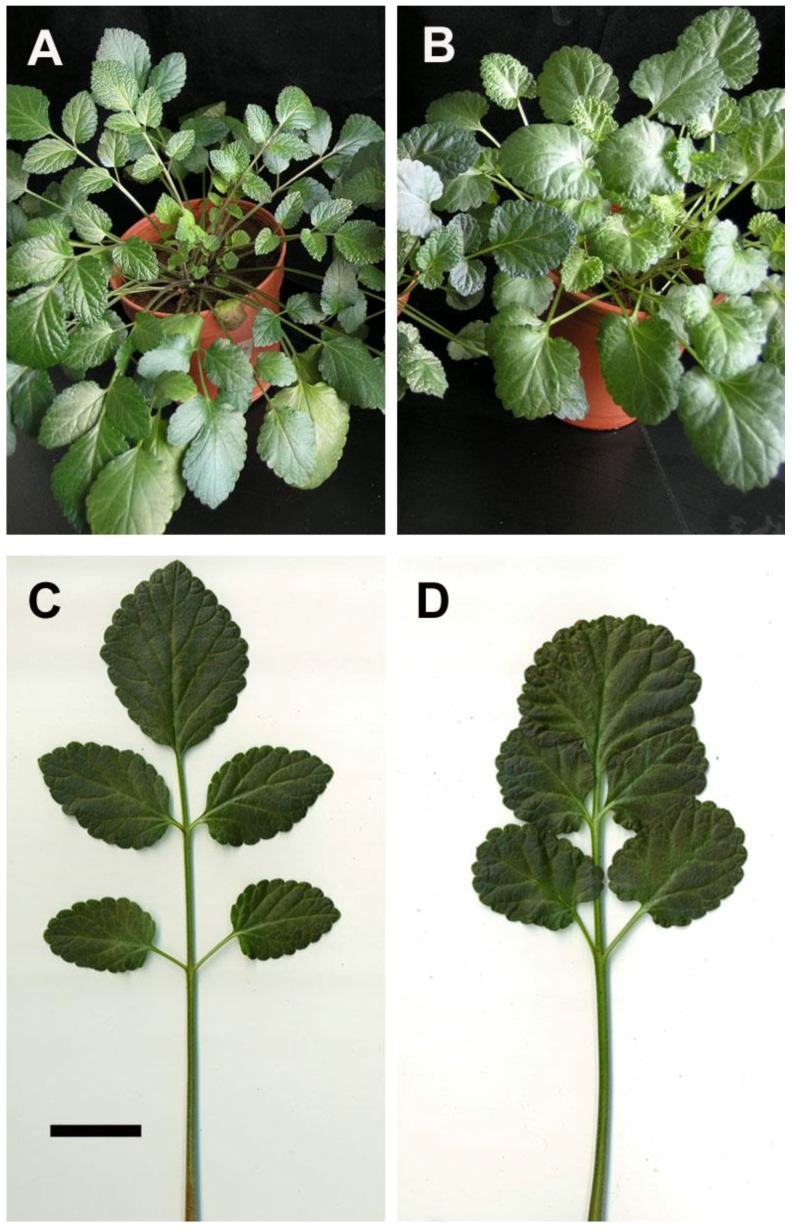
The 2*x* and 4*x* plants of *Salvia miltiorrhiza* after four months of culturing in pots (bar = 6.5 cm, 6.5 cm, 1.2 cm and 1.2 cm for Figure 5A–D, respectively). (**A**) A potted 2*x* plant. (**B**) A potted 4*x* plant. (**C**) A compound leaf of the 2*x* plant. (**D**) A compound leaf of the 4*x* plant.

After 45 days of acclimatization in plastic pots, the plants grew well with a 100% survival rate. The 4*x* plants had shorter internodes and thicker stems with broader leaves than did the 2*x* plants (Figure 5A,B). The leaflet shape of 2*x* plants was mostly ovate, but the 4*x* plants were orbiculate in the first leaflet and elliptical in the rest of the leaflets (Figure 5C,D). The petiole of the 4*x* plants was shorter and thicker than the 2*x* plants (Figure 5C,D). The leaflet length of 4*x* plants was significantly shorter than 2*x* plants, but the leaflet width and area of 4*x* plants were significant larger than 2*x* plants (Table 4).

### 2.4. Reproductive Behaviors of Ex Vitro Plants

**Figure 6:**
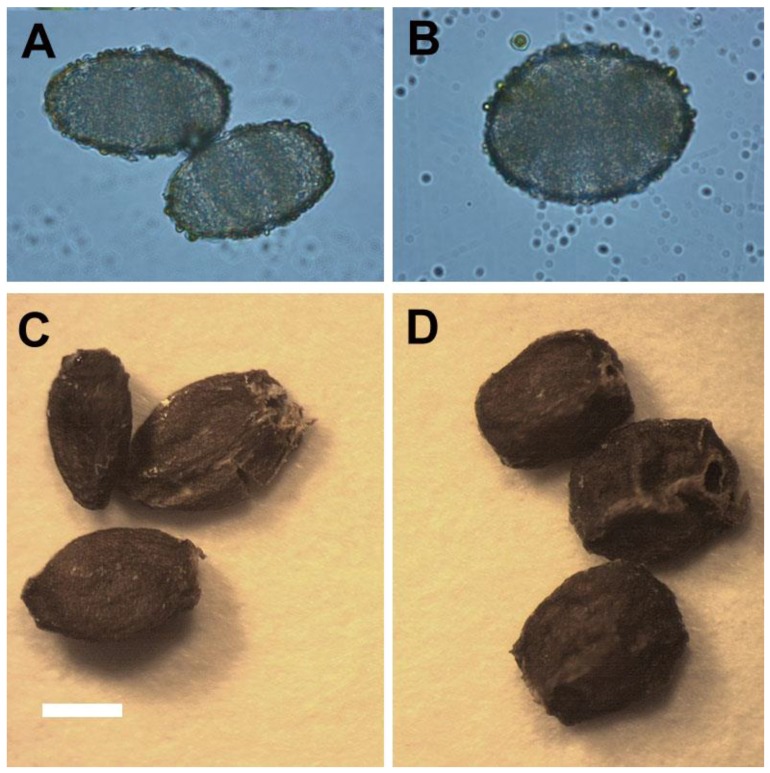
Pollens and seeds of 2*x* and 4*x* plants in *Salvia miltiorrhiza* (bar = 15 μm for all figures). (**A**) Pollens of the 2*x* plant. (**B**) Pollens of the 4*x* plant. (**C**) Seeds of the 2*x* plant. (**D**) Seeds of the 4*x* plant.

The 4*x* plants had a delay of 1–2 months in flowering when compared with 2*x* plants (data not shown). The pollen of 4*x* plants were significantly larger than 2*x* plants (Table 5, Figure 6A,B). After self-pollination for 1 month, the seeds were collected (Figure 6C,D). The seeds of 4*x* plants had a significantly greater length and width when compared with 2*x* plants (Table 5).

### 2.5. The Stability of Polyploidy

After four years of propagation in vitro using nodal stem segments from the original autotetraploids, all the 10th generation of regenerants showed a high stability in their ploidy state as indicated by flow cytometry and evaluation of selection indicators (Table 6). In addition, a highly stability of ploidy was also found in seedlings of the 4*x* plants (Table 6).

### 2.6. Evaluation of Medicinal Constituents in Ex Vitro Plants

**Figure 7:**
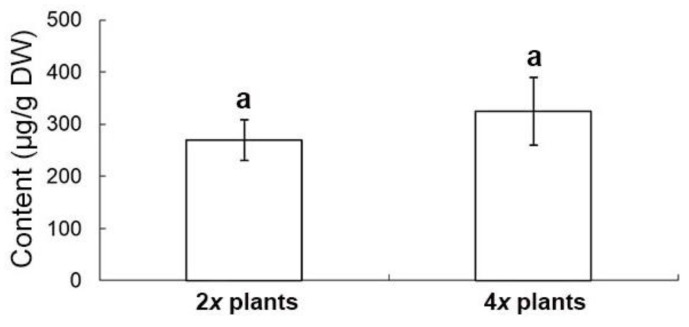
The salvianolic acid B content in shoots of 2*x* and 4*x* plants of *Salvia miltiorrhiza*. According to DMRT (*P* ≤ 0.05), the means ± standard errors that followed by the same letter were regarded as not significantly different.

**Figure 8:**
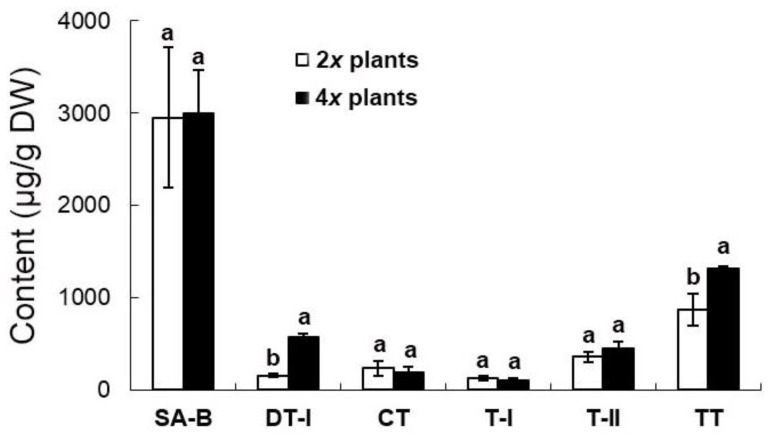
Medicinal constituents in roots of 2*x* and 4*x* plants of *Salvia miltiorrhiza*. According to DMRT (*P* ≤ 0.05), the means ± standard errors that followed by the same letter were regarded as not significantly different. CT: cryptotanshinone; DT-I: dihydrotanshinone I; T-I: tanshinone I; T-IIA: tanshinone IIA; SA-B: salvianolic acid B; TT: total tanshinones = the sum of CT, DT-I, T-I and T-IIA.

The extract from the shoots in 2*x* and 4*x* plants has only one bioactive compound, salvianolic acid B (SA-B) (Figure 7). There was no significant difference in the content of SA-B between 4*x* plants (324.4 ± 65.1 μg/g) and 2*x* plants (269.1 ± 39.1 μg/g) (Figure 7). The root of ex vitro plants accumulates not only SA-B (2952.7 ± 761.0 μg/g and 2992.0 ± 761.0 μg/g in 2*x* plants and 4*x* plants, respectively), but also four major tanshinones, including dihydrotanshinone I (DT-I) (158.0 ± 14.7 μg/g and 578.7 ± 29.5 μg/g in 2*x* plants and 4*x* plants, respectively), cryptotanshinone (CT) (234.5 ± 79.0 μg/g and 191.0 ± 58.0 μg/g in 2*x* plants and 4*x* plants, respectively), tanshinone I (T-I) (124.5 ± 25.5 μg/g and 100.0 ± 24.5 μg/g in 2*x* plants and 4x plants, respectively), and tanshinone IIA (T-IIA) (357.5 ± 57.5 μg/g and 448.5 ± 73.5 μg/g in 2*x* plants and 4*x* plants, respectively) (Figure 8). The concentration of DT-I and total tanshinones (TT, the sum of DT-I, CT, T-I and T-IIA) (874.5 ± 174.1 μg/g and 1317.8 ± 25.3 μg/g in 2*x* plants and 4*x* plants, respectively) in the root extract of 4*x* plants was significantly higher than 2*x* plants (Figure 8). In spite of the ploidy level, T-IIA was the most abundant when compared with the other three kinds of tanshinones in the root extract (Figure 8).

## 3. Discussion

The in vitro polyploidy of plants could be induced using antimitotic agents, the most commonly used being colchicine [10] The concentration and exposure time of antimitotic agents are crucial to inducing successful chromosome doubling [1,7,10]. Excessively high doses of colchicine can kill the explant or retard the growth of regenerants [1,7,9,10]. In addition, high concentrations or long exposure times for antimitotic agents can result in redoubling, causing undesirable high ploidy levels in plants [1,9,10]. A considerable lower dose of colchicine (100 mg/L) could result in a successful induction of tetraploids under the aid of TDZ-inducing organogenesis [11]. Thus, it appears that the low dosage of colchicine used in this study, prevented abnormal organogenesis and excess ploidy levels.

Explant type is a crucial factor for in vitro polyploidy induction [10]. Various explant types have been used to induce polyploidy in medicinal plants, such as callus, buds, nodes, seeds, zygotic embryos, and vegetative tissues [8,10,11,13,14,15,16,17,18,19,20]. In *S. miltiorrhiza*, the seed-derived bud clumps have been primary source of explant used in polyploidy induction [1]. In theory, seed-derived materials do not have uniform genotypes because they undergo sexual reproduction. In addition, bud clumps possess highly differentiated organs and comprise a certain amount of 2*x* cells. When the bud clumps were treated with antimitotic agents, chimeras were present because of the mixture of 2*x* with polyploidy cells.

Direct organogenesis without the intervention of dedifferentiation is, an efficient method to reduce unnecessary somaclonal variations [12,21]. In this study, the 4*x* plants of *S. miltiorrhiza* were obtained via direct organogenesis and produced stable polyploids as the 10th generation of clonal regenerants still maintained 4*x* polyploidy after four years of subculturing using nodal stem segments.

The advantages of the explant type and the regeneration pathway proposed in this study include the following: (1) The leaf explants can be obtained from uniform plantlets derived from cutting nodal stems; (2) The leaf explant can be induced to directly form adventitious shoots which is an efficient regeneration system; and (3) Chimeras can be avoided by the de novo formation of shoots from the leaf explants at the beginning of the colchicine treatments.

To determine polyploidy, several techniques have been employed, such as chromosome counting, evaluation of anatomical features, flow cytometry, and morphological comparisons [7,8,10,11,13,14,15,16,17,18,19,20]. In 63% of previous publications, flow cytometry was used to evaluate polyploidy [10]. Gao et al. used chromosome counting and morphological observation to evaluate the polyploids of *S. miltiorrhiza* [1]. These methods are laborious because they involve specific enzymatic treatments and microscopy [10]. In this study, the use of flow cytometry to evaluate polyploidy was very effective. In addition, the young leaf tissues for flow cytometric analysis can be obtained from plantlets without killing the parent plant.

Gao et al. reported an increase of the tanshinone contents in the polyploidy danshen root, but the two major compounds, DT-I and SA-B, were not determined [1]. In the extracts of danshen roots, DT-I is important in the treatment of breast cancer [22], and it had been reported as potentially valuable bioactive compound in the therapy of colorectal cancer [23]. The other compound, SA-B, was also found in the root and the potent therapeutic effects which are mainly for cardiovascular diseases [4]. It has been reported that tanshinones are synthesized via the terpenoid pathway, whereas salvianolic acids through the phenylpropanoid pathway [24]. In this study, the root extract of 4*x* plants accumulated significantly higher concentration of DT-I than 2*x* plants. However, there were no significantly difference in the concentrations of SA-B, T-I, T-IIA and CT. Altogether, this suggests the ploidy level made a contribution to the promotion of total tanshinone production mainly via DT-I biosynthesis. In *Salvia* spp., tanshinones are the major bioactive terpenoids which play important roles in growth and development of plants [25]. Consequently, in this study, several agronomic traits of 4*x* plants were also found to be enhanced, including shoot length, root diameter, number of leaves, and fresh weight of plants.

For selection of polyploidy, the increased leaf size is a useful morphological indicator [11,26]. Sugiyama demonstrated that tetraploids of *Lolium* cultivars had longer leaves with longer mature cells due to a faster rate of cell elongation than did diploids [27]. However, in this study, the leaflet of tetraploids not only had greater length, but also had greater width and area than did diploids. The ratio of leaflet length to width in tetraploids was approximately 1.21 (length/width = 3.64/3.01) which was lower than diploids with a ratio of 1.54 (length/width = 3.75/2.43). In addition, the shape of leaflet was dramatically changed by polyploidy level, and the leaflet of 4*x* plants of *S. miltiorrhiza* showed an orbiculate or elliptical shape rather than the 2*x* plants that possessed ovate leaflets. The considerable decrease of the ratio of length to width caused by polyploidy level was also found in pollen (from 1.42 to 1.30) and seeds (from 1.62 to 1.28). It was found that the pollen structures became more spherical or rounded. The ratio of length to diameter in roots was also dramatically decreased from 10.88 (2*x* plant) to 4.09 (4*x* plant), and thus the 4*x* plants had shorter and thicker roots than 2*x* plants. In previous reports, the stomatal morphology had been suggested to be a reliable indicator for selection of polyploids [26,28]. The size and the ratio of length to width (from 0.78 to 0.93) of stomata were both considerable increased in the 4*x* plants of *S. miltiorrhiza*. Therefore, the stomatal morphology was proposed as a reliable selection indicator for polyploidy in *S. miltiorrhiza*.

## 4. Materials and Methods

### 4.1. Plant Source

The initial plants, *Salvia miltiorrhiza* Bunge (2*n* = 2*x*), were purchased from Winpower Technology Co. (Kaohsiung, Taiwan). Three-month-old in vitro plantlets which have 3–5 nodes and 10–15 leaves were selected as the plant source in this study.

### 4.2. Obtaining the Donor Plants

Nodal stem segments (each was 1 cm in length) were taken from the plant source and cultured on a rooting medium to obtain donor plants for experiments. The rooting medium contained MS (Murashige and Skoog 1962) [29] medium plus 30 g/L sucrose, 1 g/L peptone, 0.5 mg/L IBA and 3 g/L Gelrite. A Magenta GA7^TM^ vessel (77 × 77 × 97 mm^3^, Merck KGaA, Darmstadt, Germany) was used as the culture container. The pH was adjusted to 5.8 with 1N HCl or 1N KOH. Each culture container contains 0.1 L of the medium and were autoclave at 15 psi and 121 °C for 20 min. The subculture period is approximately 1.5 months in a growth chamber with the culture conditions, including photoperiod, irradiance and temperature as in Tsai et al. [12].

### 4.3. Polyploidy Induction via Direct Shoot Formation

Leaf segments (approximately 2 cm^2^) were used as explants for testing the effects of colchicine on polyploidy induction. The explants were taken from plantlets which had been cultured for approximately 2 to 3 months. According Tsai et al., the highest number of shoots were obtained from leaf explants at 0.5 mg/L TDZ via direct organogenesis [12]. Therefore, various concentrations of colchicine (i.e., 0, 0.5, 1, 2, 3, 4, 5, 10, 50, and 100 mg/L) were added to 0.5 mg/L TDZ-containing MS medium for the polyploidy shoot induction experiments. In addition, a PGR-free control and a 0.5 BA-containing media were also tested to compare their effects with 0.5 mg/L TDZ. Following 3 weeks of induction, all of the cultures were transferred to the same medium that was devoid of colchicine. After an additional 6 weeks of culture, they were transferred to a medium supplemented with 0.5 mg/L BA and 0.5 mg/L IBA for 5 weeks to obtain plantlets. Subsequently, the plantlets were cultured on an MS medium supplemented with 0.5 mg/L IBA for further growth. The leaf explants were initially incubated in petri dishes in darkness for nine weeks, and thereafter, the regenerants were transferred to culture vessels in light for further growth and development. Three dishes each contained four explants (replicates) used in each treatment.

### 4.4. Analysis of Flow Cytometry

Young leaf tissues were used as materials for the polyploidy analysis performed using flow cytometry (CyFlow® Ploidy Analyser PAII, Görlitz, Germany). In the presence of 100 μL buffer (CyStain UV Precise P kit, Partec, Görlitz, Germany), the materials were chopped by a razor blade thoroughly and filtered by a 30 μm nylon mesh. For staining of the released nuclei, 400 μL of DAPI (Partec, Görlitz, Germany) were used. Each sample contained approximately 2000 nuclei for analysis. A reference 2C DNA content (i.e., C-value) was used in this study from young leaf tissues of *Phalaenopsis aphrodite* subsp. *formosana*.

### 4.5. Analysis of Stomata

To compare the difference of stomata characteristics between 2*x* and 4*x* plants, fully expanded leaves were used. The leaf tissue was taken from the third node of plantlets (approximately 2.5 month-old). For counting of stomata frequency, the epidermis was peeled and then observed with a light microscope (BX41, Olympus Corp., Tokyo, Japan), and the field area was 0.05 mm^2^. For evaluating the length and width of the randomly selected stomata.

### 4.6. Acclimatization

The procedure and the conditions of plantlet acclimatization were performed according to Tsai et al. [12]. Four-month-old pot plants were used to determine the difference in length, width and shape of the leaf. The pollens of flowering pot plants and subsequently the seeds obtained via self-pollination were collected to test their difference in size and morphology.

### 4.7. Evaluation of Ploidy Stability

Several selection indicators (stomata frequency, length of stomata, width of stomata and leaflet shape) and ploidy level were evaluated on 10th generation clonal plants (eight-month-old) obtained via in vitro nodal stem cultures and seed-derived plants obtained via self-pollination of 4*x* plants, to assess the stability of ploidy level.

### 4.8. Evaluation of Bioactive Compounds

The procedure for determination of bioactive compounds was performed as in Tsai et al. [12]. Root and shoot tissues were taken from ex vitro pot plants after 6 months of culture. The materials were harvested separately for evaluation. Bioactive compounds, including salvianolic acid B, cryptotanshinone, dihydrotanshinone I, tanshinone I, tanshinone IIA, and the total tanshinone (the sum of four tanshinone species) were assayed to compare the difference between 2*x* and 4*x* plants.

### 4.9. Statistical Analysis

A randomized complete block design was used in this study. Data evaluation in all the experiments was according to Analysis of variance (ANOVA), and each treatment contained at least four replicates (explants). The significant differences among the treatments were compared using the Duncan multiple range test [30] with a 0.05 level of probability.

## 5. Conclusions

This study successfully established an efficient and reliable protocol for inducing tetraploids in *S. miltiorrhiza* using very low dosage colchicine. The high stability of polyploidy was shown by flow cytometric analysis on four-year-old regenerants and also seedlings of autotetraploids. The resulting tetraploids showed enhanced traits in growth and tanshinone contents. These results provide an efficient platform to aid breeding programs and provide materials for further genetic studies in *S. miltiorrhiza.*

## Figures and Tables

**Table 1:** Effects of colchicine on shoot formation and subsequent polyploidy induction from the leaf explants of *Salvia miltiorrhiza* in the presence of *N^6^*-benzyladenine (BA) and 1-Phenyl-3-(1,2,3-thiadiazol-5-yl)-urea (TDZ).

Colchicine (mg/L)	0.5 mg/L BA		0.5 mg/L TDZ	
	Number of Shoot/Explants	Polyploidy (%)	Number of Shoot/Explants	Polyploidy (%)
0	9.3 ± 2.5 ^a^	0	33.3 ± 1.5 ^a^	0
0.5	3.7 ± 0.6 ^b^	9.1	13.7 ± 1.5 ^b^	39.0
1	1.3 ± 0.6 ^c^	0	11.0 ± 1.0 ^c^	18.2
2	0^c^	0	5.0 ± 1.0 ^d^	13.33
3	0^c^	0	1.3 ± 0.6 ^e^	0
4	0^c^	0	0.7 ± 0.6 ^e^	0

According to Duncan’s multiple range test (DMRT, *P* ≤ 0.05), the means ± standard errors within a column that followed by the same letter were regarded as not significantly different.

**Table 2:** Effect of polyploidy in biomass and growth of plantlets in *Salvia miltiorrhiza.*

Treatment	Fresh Weight (g)	Shoot Length (mm)	No. of Leaves	Root Length (cm)	Root Diameter (mm)
Diploid	5.29 ± 1.24 ^b^	2.53 ± 0.14 ^b^	15.3 ± 0.8 ^b^	8.70 ± 1.20 ^a^	0.80 ± 0.03 ^b^
Tetraploid	9.96 ± 1.62 ^a^	3.15 ± 0.17 ^a^	18.5 ± 0.9 ^a^	4.46 ± 0.87 ^b^	1.09 ± 0.04 ^a^

According to DMRT (*P* ≤ 0.05), the means ± standard errors within a column that followed by the same letter were regarded as not significantly different.

**Table 3:** Effect of polyploidy on characteristics of stomata in *Salvia miltiorrhiza.*

Treatment	Stomata Frequency (No./Microscopic Field)	Stomata Size	
		Length (μm)	Width (μm)
Diploid	29.0 ± 1.2 ^a^	11.5 ± 0.4 ^b^	14.7 ± 0.4 ^b^
Tetraploid	13.6 ± 0.4 ^b^	17.8 ± 0.5 ^a^	19.1 ± 0.2 ^a^

According to DMRT (*P* ≤ 0.05), the means ± standard errors within a column that followed by the same letter were regarded as not significantly different.

**Table 4:** Effect of polyploidy on morphology of leaflets in *Salvia miltiorrhiza.*

Treatment	Leaflet Length (cm)	Leaflet Width (cm)	Leaflet Area (cm^2^)	Leaflet Shape
Diploid	3.75 ± 0.11 ^a^	2.43 ± 0.07 ^b^	5.20 ± 0.27 ^b^	Ovate
Tetraploid	3.64 ± 0.12 ^b^	3.01 ± 0.11 ^a^	5.91 ± 0.31 ^a^	Orbiculate or elliptical

According to DMRT (*P* ≤ 0.05), the means ± standard errors within a column that followed by the same letter were regarded as not significantly different.

**Table 5:** Effect of polyploidy on size of pollens and seeds in *Salvia miltiorrhiza.*

Treatment	Pollen		Seed	
	Length (μm)	Width (μm)	Length (mm)	Width (mm)
Diploid	30.8 ± 0.7 ^b^	21.7 ± 0.7 ^b^	0.47 ± 0.01 ^a^	0.29 ± 0.01 ^b^
Tetraploid	38.9 ± 1.2 ^a^	30.0 ± 0.8 ^a^	0.49 ± 0.02 ^a^	0.38 ± 0.01 ^a^

According to DMRT (*P* ≤ 0.05), the means ± standard errors within a column that followed by the same letter were regarded as not significantly different.

**Table 6:** Evaluation of ploidy stability on vegetative clonal and seed-derived 4*x* plants of *Salvia miltiorrhiza.*

Characteristics	10th Generation Vegetative Clonal 4*x* Plants Obtained from Nodal Stem Segments	Seed-Derived 4*x* Plants Obtained via Self-Pollination
Level of ploidy (proved by flow cytometry)	4*x*	4*x*
Stoma frequency (no./microscopic field)	13.6 ± 1.1 ^a^	13.8 ± 1.1 ^a^
Stoma length (μm)	17.8 ± 0.8 ^a^	18.0 ± 1.0 ^a^
Stoma width (μm)	19.0 ± 1.9 ^a^	19.2 ± 1.5 ^a^
Leaflet shpae	Elliptical to orbiculate	Elliptical to orbiculate

According to DMRT (*P* ≤ 0.05), the means ± standard errors within a row that followed by the same letter were regarded as not significantly different.

## Abbreviations

BA: *N^6^*-benzyladenine; IBA: Indole-3-butyric acid; MS medium: Murashige and Skoog (1962) medium; PGRs: Plant growth regulators; TDZ: thidiazuron, 1-Phenyl-3-(1,2,3-thiadiazol-5-yl)-urea
